# Synthesis, Spectral, and Biological Properties of Copper(II) Complexes of Thiosemicarbazones of Schiff Bases Derived from 4-Aminoantipyrine and Aromatic Aldehydes

**DOI:** 10.1155/BCA/2006/59509

**Published:** 2006-06-01

**Authors:** Ram K. Agarwal, Lakshman Singh, Deepak Kumar Sharma

**Affiliations:** ^1^Department of Chemistry, School of Pure and Applied Sciences, University of the South Pacific, PO Box 1168, Suva, Fiji Islands; ^2^Department of Chemistry, Lajpat Rai Postgraduate College, Sahibabad 201005 (Ghaziahad), India

## Abstract

We have synthesized a novel series of Schiff bases by condensation of 4-aminoantipyrine and various aromatic aldehydes followed by reaction with thiosemicarbazide. These thiosemicarbazones are potential ligands toward transition metal ions. The reaction of copper(II) salts with 4[N-(benzalidene)amino]antipyrinethiosemicarbazone (BAAPTS), 4[N-(4′-methoxybenzalidene) amino] antipyrinethiosemicarbozone (MBAAPTS), 4[N-(4′-dimethylamino benzalidene) amino] antipyrinethiosemicarbazone (DABAAPTS), and 4[N-(cinnamalidene) amino] antipyrinethiosemicarbazone (CAAPTS) resulted in the formation of solid complexes with the general composition CuX_2_ · (H_2_O)(L)(X = Cl, Br,NO_3_,NCS, or CH_3_COO; L = BAAPTS, MBAAPTS, DABAAPTS, or CAAPTS). These complexes were characterized through elemental analysis, molecular weight, electrical conductance, infrared, electronic spectra, and magnetic susceptibilities at room temperature. Copper(II) complexes with BAAPTS and MBAAPTS were screened for antibacterial and antifungal properties and have exhibited potential activity. Thermal stabilities of two representative complexes were also investigated.

## INTRODUCTION

Thiosemicarbazones are now well established as an important class of sulfur donor ligands particularly for transition metal ions [1– 3]. This is due to remarkable biological activities observed for these compounds, which has since been shown to be related to their metal complexing ability. These compounds present a great variety of biological activity ranging from antitumour, fungicide, bactereocide, antiinflammatory, and antiviral activities [4– 8]. We have previously examined the chelating behaviour of some NNS donor thiosemicarbazones having pyrazolone ring in several metal complexes with the object of gaining more information about their nature of coordination and related structural and spectral properties [9, 10].

**Figure 1:**
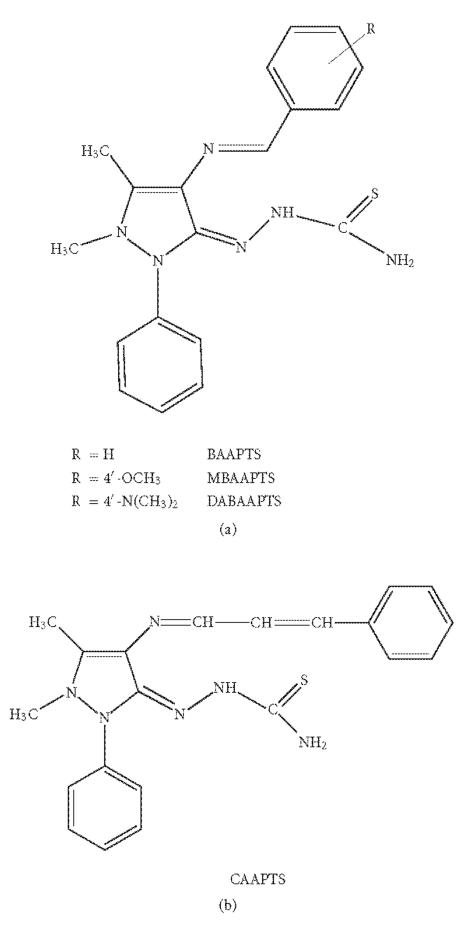
Structures of different thiosemicarbazones.

In the present work, we report the synthesis, magne-tospectral, antibacterial, and antifungal properties of copper(II) complexes of 4[N-(benzalidene) amino] antipyrinethiosemicarbazone (BAAPTS), 4[N-(4′-methoxybenzalidene) amino] antipyrinethiosemicarbazone (MBAAPTS), 4[N-(4′-dimethylaminobenzalidene) amino] antipyrinethiosemicarbazone (DABAAPTS) and 4[N-(cinnamalidene) amino] antipyrinethiosemicarbazone (CAAPTS) (Figure 1).

## EXPERIMENTAL

### Materials

Copper(II) salts, for example, copper(II) chloride, bromide, nitrate, or acetate, were obtained from BDH. AR-grade and were used as such. Cu(SCN)_2_ was prepared by mixing copper chloride (in ethanol) and ethanolic solution of potassium thiocyanate in 1 : 2 molar ratio. Precipitated KCl was filtered off, and the filtrate having copper(II) thiocyanate was used immediately for complex formation. All the four thiosemicarbazones were prepared in the laboratory by reported procedure [11]. All solvents obtained commercially were distilled before use.

The antibacterial activities of both thiosemicarbazones, that is, BAAPTS and MBAAPTS complexes of copper(II), were studied by the usual cup-plate-agar-diffusion method [12, 13]. The compounds were screened for their antibacterial activity against the following microorganisms: (a) gram positive *staphylococcus aureus* (*S aureus*), (b) gram negative *E coli*. The cup-plate-agar-diffusion method comprises of the following steps.

1. Preparation of media, sterilization, and tubing.
2. Sterilization of the cleaned glass apparatus.
3. Pouring of the seeded medium into sterilized petridishes and cutting of the cups.
4. Pouring of the dilute solution of the compounds into the tubs.
5. Incubation at a particular temperature.
6. Determination of the “zones of inhibition.”

The composition of the test media is the factor, which often exerts the greatest effect upon the drug activity. This is particularly true for thiosemicarbazones, since inhibitors of these compounds appear to be present in the common bacteriological culture medium. Efficient media of known chemical composition are available for many species such as *S aureus* and *E coli*. In addition to the composition of the test media, its pH is a factor which may directly or indirectly influence the activity of a drug. The pH of the test media taken for *S aureus* and *E coli* was adjusted in the range 7.6 ± 0.1. The composition of the basal media used in the experiments was (i) sodium chloride = 6.0 gm, (ii) peptone = 10.0 gm, (iii) beef extract = 3.0 gm, (iv) yeast extract = 2.0 gm, (v) sucrose = 1.5 gm, (vi) agar-agar = 3.0%, and (vii) distilled water = 1.0 litre.

### Procedure

The measured quantity of the culture of the test organism (0.5 mL) was added to each heated (nearly ∼ 55°C) agar-media tubes. The tubes were shaken well, and the inoculated media were poured on to the sterilized petridishes and then allowed to set in a refrigerator maintained at 4–8°C. The test solutions of 500 μg/mL and 1000 μg/mL dilutions of the respective thiosemicarbazones were prepared in a mixture of DMF and H_2_O (3 : 7, v/v). Five cups of 5 mm diameter were cut in the culture media on the pertidishes. A compound solution of particular dilution (500 μg/mL or 1000 μg/mL) was put in the outer four cups of one of the petridishes, and the second solution was put in the four cups of other petridishes. The central cups of all the petridishes were filled with the controlled solution, and all the petridishes were allowed to remain in the refrigerator maintained at ∼ 10°C for ∼ 1 hr to allow diffusion of the solution. The petridishes were then transferred to an incubator maintained at ∼ 35°C and kept for nearly 30 hrs. The zones of inhibition formed were measured with calipers. The control of DMF and H_2_O (3 : 7, v/v) showed no activity. The activity of the compounds are represented by size of the diameter in mm. The antifungal activity of the compounds was screened by using filter paper disc diffusion method. The tests were carried out by taking 6 mm diameter filter paper discs against the fungi (*A niger* and *C albicans*).

### Synthesis of the complexes

All the copper(II) complexes were synthesized by following general procedure. The corresponding copper(II) salt and the appropriate thiosemicarbazone in equimolar ratio were dissolved, separated in ethanol, and mixed together. The reaction mixture was boiled under refluxing state for ∼ 4 hrs. On cooling at room temperature, a microcrystalline complex was separated. It was filtered under suction, and the crystals were washed with cold ethanol and finally with anhydrous diethyl ether and kept in a desiccator over fused CaCl_2_.

### Analyses

Copper contents of the complexes were estimated complexometrically with EDTA using murexide and erichrome black T as an indicator after decomposing the complexes with concentrated H_2_SO_4_ and H_2_O_2_ [14]. The halogens were estimated by Volhard's method [15]. The thiocyanate was estimated by titrating slightly acidic solution of the complex with standard silver nitrate solution. Sulfur was estimated gravimetrically as BaSO_4_. The percentage of nitrogen was determined by Kjeldahl method. The molecular weight of the complexes was determined in the laboratory cryoscopically in freezing nitrobenzene using a Beckmann thermometer of accuracy ±0.01°C. The conductivity measurements were carried out using a Toshniwal conductivity bridge type CL 01/01 and dip type cell operated at 220 volts, AC mains. All the measurements were done at room temperature in PhNO_2_. The magnetic measurements on powder form of the complexes were carried out at room temperature on Gouy's balance using anhydrous copper sulfate as calibrant. The infrared spectra of the complexes were recorded on a Perkin Elmer Infrared Spectrophotometer model-521 in KBr/CsI in the range of 4000–200 cm^−1^ at the University of Delhi, Delhi, India. Diffused reflectance spectra of the solid compounds were recorded on a Beckmann-DK-2A Spectrophotometer at the University of Delhi. Thermogravimeteric analysis of the complexes was carried out in static air with open sample holder and a small platinum boat, the heating rate was 6°C/min.

## RESULTS AND DISCUSSION

The reaction of Cu^2+^ salts with BAAPTS, MBAAPTS, DABAAPTS, and CAAPTS gave complexes of the general composition CuX_2_ (L) (H_2_O) (X = Cl, Br, NO_3_, NCS, or CH_3_COO; L = BAAPTS, MBAAPTS, DABAAPTS, or CAAPTS). The analytical data of these complexes are presented in Table 1. All the complexes are quite stable and could be stored for months without any appreciable change. The complexes do not have sharp melting point, but decomposed on heating beyond 250°C. These complexes are generally soluble in common organic solvents. The molar conductance values of the complexes in PhNO_2_ are presented in Table 1. The values are too low to account for any dissociation, therefore the complexes are considered to be nonelectrolytes [16]. The molecular weights determined by cryoscopic method in PhNO_2_ (Table 1) are in broad agreement with the conductance data. The observed magnetic moments of all these complexes (Table 1) are in 1.81–1.92 BM range. The observed magnetic moments of the complexes are consistent with the presence of a single unpaired electron [17, 18].

### Biological properties

A number of authors [19– 23] were interested to investigate the biological and medicinal properties of transition metal complexes of thiosemicarbazones. Thomas and Parmeswaran [20] studied the antitumour activities of Mn^2+^, Co^2+^, Ni^2+^, and Cu^2+^ chelates of anthracene-9-carboxaldehyde thiosemicarbazone. Murthy and Dharmaraja [21] reported the cytotoxic activity of phenylglyoxal bis(thiosemi-carbazone) against *Ehrlich ascites* carcinoma cells. These compounds were also screened for antimicrobial activity on *B subtilis* and *E coli*. They inhibited the bacterial growth considerably. Garg et al [24] have recently reported the antifungal activity of some transition metal complexes of 2-(2′-hydroxybenzylidene)aminophenyl benzimidazole. All complexes were screened against *Alternaria alternata* and *Aspergillus niger* by spore germination inhibition method at concentrations 100, 500, and 1000 ppm using Dithane M-45 as a standard. Recently, Singh [25] published a review article on metal complexes of glutathione and their biological properties. Copper(II) is the most important oxidation state of copper in many physiological systems. Cu(II) complexes of glutathione were tested for their antifungal activity against some plant pathogenic fungi using slide germination technique [26]. Cu(I) is another important oxidation state of copper in physiological systems. Cu(I)-thioamino complex formation serves not only to improve the chelation therapy for treating intoxication, but may also provide a better understanding of many facets of normal copper metabolism [27–29], since copper is an essential trace metal which can adopt a redox system in biological system allowing it to play a pivotal role in physiology. Copper homeostasis in biological system is well characterized, involving several proteins such as glutathione, metallothione, ATPase, Menkes, and Wilso proteins as well as the cytoplasmic copper chaperons. In view of the biological relevance of copper(II) complexes, in the present studies, the antibacterial activities of the copper(II) complexes of BAAPTS and MBAAPTS and standard drugs (ampicillin and teracycline) were screened by the agar-cup method in DMF solvent at a concentration of 50 μg/mL and were checked against gram positive bacteria *B subtilis* and *S aureus* and gram negative bacteria *E coli* and *S typhi* (Table 2). Diameters of zone of inhibition (in mm) of standard drug ampicillin against gram positive bacteria *B subtilis* and *S aureus* and gram negative bacteria *E coli* and *S typhi* were found to be 15, 13, 17, and 18, respectively, while tetracycline gave 18, 17, 21, and 22, respectively, under identical conditions, Table 2 show that all copper(II)-thiosemicarbazone complexes have moderate antibacterial activities against these bacteria. Both thiosemicarbazones and their copper(II) complexes were screened for their antifungal activities against two fungi (*A niger* and *C albicans*). The results (Table 2) showed that almost all complexes showed nearly the same extent of activity, but they are less active compared to salicylic acid. It is interesting to note that due to the presence of methoxy group and comparatively faster diffusion of MBAAPTS complexes, they showed increased activity than that of BAAPTS complexes. These compounds were found to be efficient antifungal agents.

## INFRARED

A study and comparison of infrared spectra of free ligands (BAAPTS, MBAAPTS, DABAAPTS, or CAAPTS) and their Cu^2+^ complexes (Tables 3 and 4) imply that these ligands behave as neutral tridentate and the copper(II) is coordinated through N & N of two azomethine groups and of S of thio-keto group.

The strong bands observed at 3440–3270 cm^−1^ region in the free ligands have been assigned to *ν*(NH) vibrations. Practically no effect on these frequencies after complexation precludes the possibility of complexation at this group. The absorption at ∼ 1600 cm^−1^ in the free ligands can be attributed to (C=N) stretching vibrations of imine nitrogen, which is in agreement with the observations of previous authors [30, 31]. On complexation, these frequencies were observed to be shifted to lower wave number (Tables 3 and 4). These observations suggest involvement of unsaturated nitrogen atoms of the two azomethine groups in bonding with the metal ion. In substituted thioureas, the (C=S) stretching vibrations are contributed much with some other vibrations as (CN) stretching and bending as well as (N−C−S) bending modes [32]. In the spectra of the present ligands, the bands observed in 1330–1305 cm^−1^ region, 1120–1095 cm^−1^, and 820–760 cm^−1^ regions are assigned to [*ν*(C=S)+*ν*(C=N) + *ν*(C−N)], [*δ*(N−C−S) + *δ*(C=S)] bending and *ν*(C = S−) stretching, respectively, following the observations of Irving et al [33] and some other authors [34, 35]. Coordination of sulfur with metal ion would result in the displacement of elections toward the latter, thus resulting in the weakening of (C=S) bond. Hence, on complexation, (C=S) stretching vibrations should decrease and that of (CN) should increase [35, 36]. In all present complexes of Cu^2+^ with BAAPTS, MBAAPTS, DABAAPTS, and CAAPTS, the frequencies in 1330–1305 cm^−1^ get an increase by nearly 30–60 cm^−1^. Similarly, bending modes of (N−C−S) and (C=S) also get increase, but in lesser amount. On the other hand, on complexation, the frequencies in 820–760 cm^−1^ are shifted to lower wave numbers and intensity of the bands are also reduced. All these peculiar changes on complexation confidently precludes any unambigious ascertain of metal-sulfur bond.

The possibility of thione-thiol tautomerism (H−N−C=S) (C=N−SH) in these ligands has been ruled out for no bands around 2700–2500 cm^−1^, characteristic of thiol group is displayed in the infrared absorption [37, 38]. In far infrared region, the bands in 410–330 cm^−1^ are tentatively assigned to *ν*(Cu−N)/*ν*(Cu−N) (metal-ligand) stretching bands [39, 41]. In conclusion, the infrared spectral studies suggest the tridentate (N,N,S) nature by pointing out the sites of possible donor atoms.

The presence of coordinated water was suggested by the very broad absorption centered around 3450 cm^−1^ in the infrared spectra. Bands at ∼ 930 and 770 cm^−1^ may be attributed to rocking and wagging modes of the coordinated water [42].

## ANIONS

The pseudohalide (SCN^−^) ion is a very interesting anion since it may coordinate through the sulfur (thio-) or through the nitrogen (isothio-) or through both these atoms (bridging). The various criteria proposed for determining the mode of bonding have been discussed by Nakamoto [42]. In general, the bonding depends on (a) the nature of the central atom, (b) the nature of other ligands in the coordination sphere, and (c) environmental controls and kinetic (mechanistic) controls. In the present complexes, the frequencies in 2045–2035 cm^−1^ due to (C−N) stretch (*ν*_1_), 845–835 cm^−1^ due to *ν*(C−S) stretch (*ν*_2_), and 465–400 cm^−1^ for *δ*(NCS) have been identified. These frequencies are associated with the terminal N-bonded isothiocyanate ions [43, 44]. In nitrate complexes, the occurrence of two strong bands in 1550–1535 cm^−1^ and 1315–1300 cm^−1^ are attributed to *ν*_4_ and *ν*_1_ modes of vibrations of covalently bonded nitrate groups, respectively. This suggests that nitrate groups are present inside the coordination sphere [45, 46]. Other absorptions associated with covalently bonded nitrate groups are also observed in the spectra of these complexes. If the (*ν*_4_ − *ν*_1_) difference is taken as an approximate measure of the covalency of nitrate group [46, 47], a value of ∼ 200 cm^−1^ for the complexes studied suggests strong covalency for the metal-nitrate bonding. Lever et al [48, 49] have shown that the number and relative energies of nitrate combination frequencies (*ν*_1_ + *ν*_4_) in the 1800–1700 cm^−1^ region of the infrared spectrum, may be used as an aid to distinguish the various coordination's modes of the nitrate group. According to Lever et al [48, 49], bidentate coordination involves a greater distortion from D_3h_ symmetry than unidentate coordination, therefore, bidentate complexes should show a larger separation of (*ν*_1_ + *ν*_4_). The authors have tried to apply this method to the present complexes. In all present complexes, a separation of 15–25 cm^−1^ in the combination bands (*ν*_1_ + *ν*_4_) in the 1800–1700 cm^−1^ region concludes the monodentate nitrate coordination.

## ELECTRONIC SPECTRA

Electronic spectra data of all copper(II) complexes are collected in Table 5. The spectra of these complexes consist of a broad band (16000 ± 200 cm^−1^) of medium intensity in the visible region which can be identified as a *d − d* band of the central ion, that is, an electronic transition mainly localized on Cu(II). The spectra of tetragonality distorted complexes should consist of three bands corresponding to the transitions ^2^B_1g_→ ^2^A_2_, ^2^B_1g_→ ^2^B_2g_, and ^2^B_1g_→ ^2^E_g_ in order to increase energy. But generally, such complexes exhibit [50, 51], a broad structureless band with or without shoulder between 14000–18000 cm^−1^ depending upon the strength of in-plane and axial ligands. Since only a single *d − d* broad band at 16000 ± 200 cm^−1^ has been observed in the complexes reported herein, it is concluded that all three transitions be within this broad envelope. The calculated 10 Dq values are also included in Table 5.

## THERMAL STUDIES

Comparatively less is known about the thermal properties of transition metal complexes of thiosemicarbazones [39–41, 52–54]. In the present work, we report herein the thermal decomposition data of the two representative complexes, that is, [Cu(BAAPTS)H_2_O(NO_3_)_2_] and [Cu(CAAPTS)H_2_O(CH_3_COO)_2_], presented in Table 6. The careful analyses of thermogravimetric curves suggest that both complexes contain one molecule of coordinated water, which is evident by loss in weight at ∼ 160°C. There is no change upto ∼ 300°C after that there is a break in the curves due to evaporation of 0.5 molecule of organic ligand, the remaining ligand is removed from the coordination sphere at ∼ 600°C. Finally, at ∼ 760°C, CuO is formed [37, 38].

**Scheme 1:**
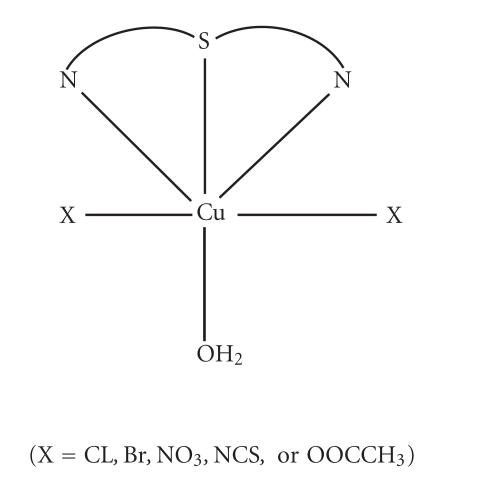
(X = Cl, Br, NO_3_, NCS, or OOCCH_3_).

The probable structures of the present complexes are suggested as in Scheme 1.

## CONCLUSION

Thus in the present studies, all the thiosemicarbazones are coordinating to Cu^2+^ ion as neutral tridentate (N, N, S) ligands. The magnetic and elecytronic spectral studies suggest the distorted octahedral geometries of the present complexes. Copper(II) complexes of BAAPTS and MBAAPTS have moderate antibacterial activity against *E coli* and *S typhi* and antifungal activity against *A niger* and *C albicans*.

## Figures and Tables

**Table 1:** Analytical, conductivity, molecular weight, and magnetic moment data of Cu^2+^ complexes of thiosemicarbazones.

Complex	Yield	Analysis : Found (Calcd) %				mw Found	Λ_*m*_	*μ*_eff_
	(%)	Cu	N	S	Anion	(Calcd)	( ohm^−1^ cm^2^ mole^−1^)	BM

CuCl_2_(H_2_O)BAAPTS	75	12.19	16.17	6.14	13.62	511	3.6	1.83
		(12.29)	(16.26)	(6.19)	(13.74)	(516.5)		
CuBr_2_(H_2_O)BAAPTS	70	10.38	13.73	5.23	26.22	599	3.7	1.92
		(10.48)	(13.87)	(5.28)	(26.42)	(605.5)		
Cu(NO_3_)_2_(H_2_O)BAAPTS	75	11.10	19.53	5.57	—	558	2.3	1.89
		(11.15)	(19.66)	(5.61)	—	(569.5)		
Cu(NCS)_2_(H_2_O)BAAPTS	70	11.19	19.79	16.92	20.47	558	3.9	1.87
		(11.30)	(19.94)	(17.09)	(20.65)	(561.5)		
Cu(OAc)_2_(H_2_O)BAAPTS	67	11.15	14.78	5.61	—	557	2.7	1.81
		(11.26)	(14.90)	(5.67)	—	(563.5)		
CuCl_2_(H_2_O)MBAAPTS	73	11.94	15.76	6.00	13.31	539	3.7	1.82
		(11.61)	(15.37)	(5.85)	(12.99)	(546.5)		
CuBr_2_(H_2_O)MBAAPTS	70	10.19	13.49	5.13	25.74	629	3.9	1.92
		(9.99)	(13.21)	(5.03)	(25.17)	(635.5)		
Cu(NO_3_)_2_(H_2_O)MBAAPTS	75	10.83	19.13	5.44	—	590	5.6	1.82
		(10.39)	(18.68)	(5.33)	—	(599.5)		
Cu(NCS)_2_(H_2_O)MBAAPTS	70	10.99	19.38	16.63	19.35	583	3.9	1.89
		(10.73)	(18.93)	(16.22)	(19.61)	(591.5)		
Cu(OAc)_2_(H_2_O)MBAAPTS	65	10.95	14.48	5.51	—	587	2.7	1.84
		(10.69)	(14.15)	(5.39)	—	(593.5)		
CuCl_2_(H_2_O)DABAAPTS	72	11.26	17.39	5.65	12.52	552	3.3	1.82
		(11.34)	(17.51)	(5.71)	(12.68)	(559.5)		
CuBr_2_(H_2_O)DABAAPTS	70	9.69	15.01	4.87	24.47	644	3.7	1.92
		(9.79)	(15.11)	(4.93)	(24.67)	(648.5)		
Cu(NO_3_)_2_(H_2_O)DABAAPTS	65	10.29	20.67	5.16	—	607	3.0	1.81
		(10.36)	(20.89)	(5.22)	—	(612.5)		
Cu(NCS)_2_(H_2_O)DABAAPTS	65	10.42	20.69	15.75	19.03	598	5.3	1.87
		(10.50)	(20.84)	(15.88)	(19.18)	(604.5)		
Cu(OAc)_2_(H_2_O)DABAAPTS	60	10.37	16.07	5.18	—	602	4.7	1.83
		(10.46)	(16.15)	(5.27)	—	(606.5)		
CuCl_2_(H_2_O)CAAPTS	73	11.94	15.76	6.00	13.31	539	3.7	1.82
		(11.61)	(15.37)	(5.85)	(12.99)	(546.5)		
CuBr_2_(H_2_O)CAAPTS	70	10.19	13.49	5.13	25.74	539	3.6	1.92
		(9.99)	(13.21)	(5.03)	25.17	(546.5)		
Cu(NO_3_)_2_(H_2_O)CAAPTS	75	10.83	19.13	5.44	—	629	2.7	1.82
		(10.39)	(18.68)	(5.33)	—	(635.5)		
Cu(OAc)_2_(H_2_O)CAAPTS	65	10.95	14.48	5.51	—	587	3.1	1.84
		(10.69)	(14.15)	(5.39)	—	(599.5)		
Cu(NCS)_2_(H_2_O)CAAPTS	70	10.99	19.38	16.63	19.35	583	3.3	1.84
		(10.73)	(18.93)	(16.22)	(19.61)	(591.5)		

**Table 2:** Antibacterial and antifungal activities of copper(II) complexes of BAAPTS and MBAAPTS.

Complexes	Antibacterial activity				Antifungal activity	

	(Zone size in mm)*	
	*Bs*	*Sa*	*Ec*	*St*	*A niger*	*C albicans*

CuCl_2_(H_2_O)(BAAPTS)	12	11	11	12	+	+
CuBr_2_(H_2_O)(BAAPTS)	11	10	11	09	+	+
Cu(NO_3_)_2_(H_2_O)(BAAPTS)	13	10	10	10	+	+
Cu(NCS)_2_(H_2_O)(BAAPTS)	13	12	11	10	++	++
Cu(CH_3_COO)_2_(H_2_O)(BAAPTS)	11	10	12	10	+	+
CuCl_2_(H_2_O)(MBAAPTS)	15	12	13	14	++	++
CuBr_2_(H_2_O)(MBAAPTS)	14	12	12	10	++	++
Cu(NO_3_)_2_(H_2_O)(MBAAPTS)	15	11	11	10	++	++
Cu(NCS)_2_(H_2_O)(MBAAPTS)	16	14	16	15	++	+
Cu(CH_3_COO)_2_(H_2_O)(MBAAPTS)	14	11	14	12	++	++
Ampicillin	24	22	17	16	—	—
Tetracycline	18	17	21	22	—	—
Salicylic acid	—	—	—	—	+ + ++	+ + ++

*Result of representative experiments.

**Table 3:** Key IR bands (cm^−1^) of BAAPTS and MBAAPTS and their Cu^2+^ complexes.

Compounds	Assignments						

	*ν*(NH)	*ν*(C=N)	*ν*(C=S) + *ν*(C+N)	δ(NCS)+ CS-	*ν*(N−N)	*ν*(C=S)	*ν*(Cu−N)/
			+ *ν*(C−N)	bending			*ν*(Cu−S)

BAAPTS	3440 s	1600 vs	1330 s	1120 m	1050 m	820 s	—
	3270 m	—	1305 s	1095 m	—	760 vs	—
CuCl_2_ (BAAPTS)H_2_O	3442 s	1575 s	1390 m	1150 m	1060 m	780 s	420 m
	3275 m	—	1345 m	1130 m	—	740 m	310 m
CuBr_2_(BAAPTS)H_2_O	3445 m	1572 s	1365 m	1160 m	1065 m	770 s	405 m
	3280 m	—	1335 m	1135 m	—	745 m	318 w
Cu(NO_3_)_2_(BAAPTS)H_2_O	3400 s	1560 s	1375 m	1160 m	1062 m	755 s	415 m
	3272 m	—	1340 m	1135 m	—	730 s	310 w
Cu(NCS)_2_(BAAPTS)H_2_O	3445 s	1565 s	1370 m	1165 m	1065 m	772 s	410 m
	3270 m	—	1345 m	1140 m	—	745 m	305 w
Cu(OAc)_2_(BAAPTS)H_2_O	3445 s	1560 s	1370 m	1155 m	1060 m	770 m	412 m
	3275 m	—	1340 m	1130 m	—	740 m	305 w
MBAAPTS	3420 s	1600 vs	1320 s	1120 m	1060 m	840 s	—
	3310 s	—	1195 m	1095 m	—	820 s	—
CuCl_2_(MBAAPTS)H_2_O	3415 m	1575 s	1365 s	1170 m	1072 m	772 s	415 m
	3312 m	—	1240 m	1130 m	—	755 s	302 w
CuBr_2_(MBAAPTS)H_2_O	3422 m	1570 s	1360 s	1172 m	1070 m	770 s	398 m
	3310 m	—	1245 m	1125 m	—	750 s	300 w
Cu(NO_3_)_2_(MBAAPTS)H_2_O	3418 m	1562 s	1362 s	1175 m	1068 m	771 s	408 m
	3310 m	—	1242 m	1130 m	—	752 s	305 w
Cu(NCS)_2_(MBAAPTS)H_2_O	3415 m	1565 s	1362 s	1172 m	1072 m	770 s	415 m
	3315 w	—	1240 m	1130 m	—	745 s	310 w
Cu(OAc)_2_(MBAAPTS)	3415 m	1568 s	1370 s	1172 m	1070 m	782 s	410 m
	3315 m	—	1245 m	1125 m	—	755 m	312 w

**Table 4:** Key IR bands (cm^−1^) of DABAAPTS and CAAPTS and their Cu^2+^ complexes.

Compounds	*ν*(NH)	*ν*(C=N)	*ν*(C=S) + *ν*(C=N)	δ(NCS) + CS	*ν*(N−N)	*ν*(C=S)	*ν*(Cu−N)/
			+ *ν*(C−N)	bending			*ν*(Cu−S)

DABAAPTS	3360 s	1600 vs	1310 s	1115	1050 m	830 s	—
	3330 m	—	1290 s	1095	—	730 s	—
CuCl_2_(DABAAPTS)H_2_O	3362 s	1572 vs	1365 m	1165	1065 m	782 m	410 m
	3320 m	—	1340 m	1132	—	710 m	305 w
CuBr_2_(DABAAPTS)H_2_O	3360 s	1570 s	1370 m	1172	1068 m	775 m	415 m
	3335 m	—	1330 m	1130	—	710 m	300 w
Cu(NO_3_)_2_(DABAAPTS)H_2_O	3360 s	1575 s	1365 m	1160	1062 m	770 m	415 m
	3332 m	—	1342 m	1142	—	705 m	302 w
Cu(NCS)_2_(DABAAPTS)H_2_O	3362 s	1565 vs	1372 s	1165	1060 m	775 m	400 m
	3330 m	—	1345 m	1130	—	722 m	305 w
Cu(OAc)_2_(DABAAPTS)H_2_O	3365 s	1565 s	1370 s	1162	1065 m	770 m	410 m
	3335 m	—	1340 m	1135	—	715 m	310 w
CAAPTS	3315 s	1605 vs	1320 s	1125	1060 m	840 m	—
	3200 m	—	1295 s	1090	—	770 m	—
CuCl_2_(CAAPTS)H_2_O	3320 s	1555 s	1370 m	1180	1070 m	790 m	415 m
	3205 m	—	1330 m	1140	—	755 m	302 w
CuBr_2_(CAAPTS)H_2_O	3315 s	1565 vs	1365 s	1175	1068 m	795 m	410 m
	3202 m	—	1325 m	1135	—	750 m	335 w
Cu(NO_3_)_2_(CAAPTS)H_2_O	3315 s	1572 s	1375 s	1170	1072 m	792 m	402 m
	3205 m	—	1335 m	1130	—	745 m	305 w
Cu(NCS)_2_(CAAPTS)H_2_O	3312 s	1570 s	1360 m	1172	1075 m	780 m	415 m
	3200 m	—	1330 m	1125	—	748 m	300 w	
Cu(OAc)_2_(CAAPTS)H_2_O	3315 s	1570 s	1355 m	1170	1070 m	795 m	408 m
	3205 m	—	1320 m	1130	—	750 m	303 w

**Table 5:** Electronic spectral bands (cm^−1^) of Cu^2+^ complexes of thiosemicarbazones.

Complex	(*d − d*) band	CT-bands		10 Dq

CuCl_2_(BAAPTS)H_2_O	15800	23000,	28700	7900
CuBr_2_(BAAPTS)H_2_O	16250	23500,	28600	8125
Cu(NO_3_)_2_(BAAPTS)H_2_O	16100	22900,	28700	8050
Cu(NCS)_2_(BAAPTS)H_2_O	16200	23200,	28700	8100
Cu(OAc)_2_(BAAPTS)H_2_O	16300	23000,	28800	8150
CuCl_2_(MBAAPTS)H_2_O	16000	23000,	26600	8000
CuBr_2_(MBAAPTS)H_2_O	16000	23000,	26900	8000
Cu(NO_3_)_2_(MBAAPTS)H_2_O	16300	23000,	28700	8150
Cu(NCS)_2_(MBAAPTS)H_2_O	16000	23000,	28850	8000
Cu(OAc)_2_(MBAAPTS)H_2_O	16100	22900,	28750	8050
CuCl_2_(DABAAPTS)H_2_O	15800	23100,	28770	7900
CuBr_2_(DABAAPTS)H_2_O	16060	23080,	28700	8030
Cu(NO_3_)_2_(DABAAPTS)H_2_O	16000	23000,	28750	8000
Cu(NCS)_2_(DABAAPTS)H_2_O	16150	23010,	26900	8075
Cu(OAc)_2_(DABAAAPTS)H_2_O	15800	23080,	28770	7900
CuCl_2_(CAAPTS)H_2_O	16300	23200,	28700	8150
CuBr_2_(CAAPTS)H_2_O	16200	23200,	28700	8100
Cu(NO_3_)_2_(CAAPTS)H_2_O	15800	22900,	28700	7900
Cu(NCS)_2_(CAAPTS)H_2_O	16150	23000,	26600	8075
Cu(OAc)_2_(CAAPTS)H_2_O	16200	23100,	28700	8150

**Table 6:** Thermal decomposition data for Cu^2+^ complexes of BAAPTS and CAAPTS.

Complex	Stage	Reaction	Peak	Temp	Peak
	of		temp (°C) in	range in	temp (°C)
	decomposition		dtg	dtg (°C)	in dta

Cu(BAAPTS)H_2_O(NO_3_)_2_	I	Cu(BAAPTS) H_2_O (NO_3_)_2_ → Cu(BAAPTS)(NO_3_)_2_	140	105–170	150 (endo)
	II	Cu(BAAPTS)(NO_3_)_2_ → Cu(BAAPTS)_0.5_(NO_3_)_2_	340	295–380	335 (exo)
	III	Cu(BAAPTS)_0.5_(NO_3_)_2_ → Cu(NO_3_)_2_	540	500–590	525 (exo)
	IV	Cu(NO_3_)_2_ → CuO	730	685–770	740 (exo)

Cu(CAAPTS)H_2_O(OAc)_2_	I	Cu(CAAPTS)H_2_O(OAc)_2_ → Cu(CAAPTS)OAc)_2_	150	120–160	135 (endo)
	II	Cu(CAAPTS)(OAc)_2_ → Cu(CAAPTS)_0.5_(OAc)_2_	350	310–395	360 (exo)
	III	Cu(CAAPTS)_0.5_(OAc)_2_ → Cu(OAc)_2_	550	510–600	535 (exo)
	IV	Cu(OAc)_2_ → CuO	710	670–755	720 (exo)
